# Correction of Single-Tooth Crossbite in Children: A Report of Three Cases

**DOI:** 10.7759/cureus.88086

**Published:** 2025-07-16

**Authors:** Shreyas Neelkanthan, Arati Vaiude, Shivani Dhonde, Ashwin M Jawdekar, Laresh N Mistry, Sharvari Pokale

**Affiliations:** 1 Department of Pediatric and Preventive Dentistry, Bharati Vidyapeeth (Deemed to be University) Dental College and Hospital, Navi Mumbai, IND; 2 Department of Periodontology, Bharati Vidyapeeth (Deemed to be University) Dental College and Hospital, Navi Mumbai, IND

**Keywords:** anterior crossbite, early orthodontic intervention, hawley's appliance, interceptive orthodontics, laser frenectomy, removal appliance with z-spring, tongue blade therapy, z-spring

## Abstract

Single-tooth anterior dental crossbite is a commonly observed malocclusion in the mixed dentition of children. Early intervention is crucial since malocclusion does not correct itself and can contribute to enamel wear, periodontal disease, and disturbances in temporomandibular joint function. Several treatment methods have been proposed to correct it, including removable appliances and uncomplicated interceptive techniques. This case report shows three pediatric patients diagnosed with single-tooth anterior crossbite. In one case, the crossbite was successfully treated with a Hawley's appliance featuring a Z-spring and a posterior bite plane in just a few weeks. In the remaining two cases, tongue blade therapy was used as a minimally invasive treatment. In one case, crossbite correction was achieved following the performance of a diode laser-assisted frenectomy, which was necessary because a thick labial frenum was obstructing the proper alignment of the teeth.

## Introduction

Anterior single-tooth crossbite is a common malocclusion observed during the mixed dentition period, with a reported prevalence of approximately 26.7% in school children [1]. According to Alaoui and Chhoul (2019), the prevalence ranged from 2.2% to 36% [2]. If left untreated, it may lead to harmful effects, including enamel abrasion, periodontal issues, and temporomandibular joint dysfunction [3]. Crossbites are classified according to their site (anterior or posterior) and etiology (skeletal, dental, or functional) [1]. Anterior dental crossbite is a discrete malocclusion affecting one or more teeth. Early treatment is crucial for preventing the development of these complications and guiding the developing dentition toward a stable occlusion. Numerous treatment modalities have been reported for correcting single-tooth anterior crossbites [4]. Removable devices, such as Z-springs, in conjunction with a posterior bite plane, have been employed in carefully selected cases. Catlan’s appliance, a lower anterior inclined plane, has also been recommended explicitly in tipping-type cases and has been found to yield satisfactory results during the early mixed dentition phase [5,6]. Fixed appliances, such as 2 × 4 sectional brackets with light continuous force archwires, are generally accepted in the treatment of single-tooth anterior crossbites, particularly where controlled rotation or root movement is needed [7]. The selection of the appliance is highly based on the etiology and specific needs of the case. Correction in the mixed dentition stage has less need for extensive treatment, avoids malocclusion of permanent dentition, and has long-term implications. The use of space maintainers, habit-breaking appliances, and removable functional appliances is one method employed in interceptive orthodontics. Interceptive orthodontics, a boon, has its limitations, including patient compliance and unpredictable growth patterns [8]. Some of the suggested treatment methods by researchers include Hawley's retainers with Z-springs and posterior bite planes, tongue blade therapy, labial and lingual archwires, Catlan's appliance (a lower inclined bite plane), and crowns fabricated using composite or stainless steel. One popular and successful method for correcting a single-tooth anterior crossbite is Hawley's appliance with a Z-spring and a posterior bite plane, a less intrusive, comfortable, and effective procedure in children [9].

The Z-spring, made of stainless steel wire, imparts a labial force to restore the impacted maxillary incisor to its normal position. The posterior bite plane acts to temporarily exclude the front teeth, thereby eliminating occlusal interference and allowing for free movement of the displaced tooth. Tongue blade therapy is another procedure where patients are instructed to place the tongue blade on the palatal surface of the maxillary incisors and bite softly, creating a forward-directed force that guides the maxillary incisor into its correct position [10]. This case series aims to illustrate the diagnosis and treatment of a single-tooth anterior crossbite using Hawley's appliance with a Z-spring, a posterior bite plane, and tongue blade therapy, highlighting the clinical decision-making process and treatment outcomes.

## Case presentation

Case 1

A 12-year-old boy reported to the Department of Pediatric and Preventive Dentistry with the chief complaint of misaligned upper front teeth. His family and medical histories were non-contributory, and no deleterious oral habits were seen. On intraoral examination, he was found to be in the permanent dentition stage, and the maxillary right central incisor was found to be positioned palatally, which caused an anterior crossbite (Figure 1). Upon clinical examination, a diagnosis of Angle's Class I malocclusion with an anterior crossbite of tooth number 11 was made. Although a meticulous history was taken to investigate the probable etiology of the crossbite, it could not be identified definitively. Regarding model analysis, due to the phase of dentition, a comprehensive orthodontic analysis is planned for a subsequent phase, when all necessary analyses will be performed. Options for treatment were explained in detail to the patient's parents, and informed consent was obtained before initiating care.

**Figure 1:**
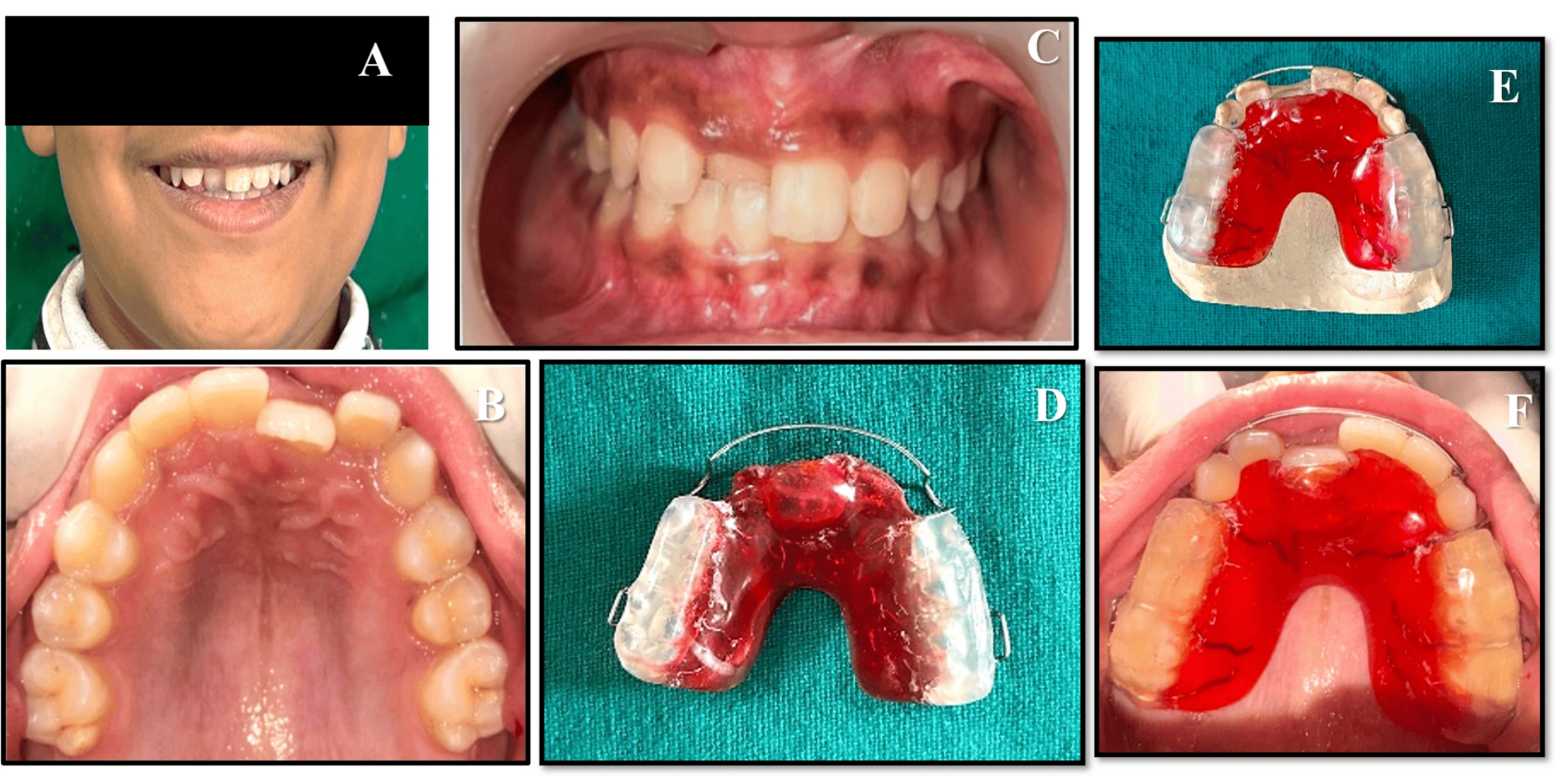
Pre-treatment records (A) Extraoral photograph of the patient smiling. (B) Pre-treatment photograph of maxillary arch. (C) Pre-treatment photograph at centric occlusion. (D) Hawley’s appliance with a Z-spring and posterior bite plate. (E) Hawley’s appliance on the study model. (F) Maxillary arch with Hawley’s appliance intraorally.

Treatment Plan

On the first visit, following parents' consent, the pre-treatment records were taken, including intraoral and extraoral photographs (Figure 1) and alginate impressions of the maxillary and mandibular arches. On the second visit, oral prophylaxis was performed. Hawley's appliance, featuring a Z-spring and posterior biteplate, was constructed (Figure 1). Adam's labial bow and clasp were the retentive elements of the appliance, constructed from 21-gauge round stainless steel wire.

The appliance was delivered with proper instructions, and the Z-spring coil was activated following delivery. It was then periodically activated by opening the spring during the subsequent few visits, and the patient was seen weekly to check progress. Following repeated activations, the desired correction of the anterior crossbite was attained (Figure 2). A retainer was not found to be necessary after the correction of the single-tooth anterior crossbite, since cases such as this one usually become stable by themselves. The result effectively addressed the patient's aesthetic needs and alleviated the parents' concerns.

**Figure 2:**
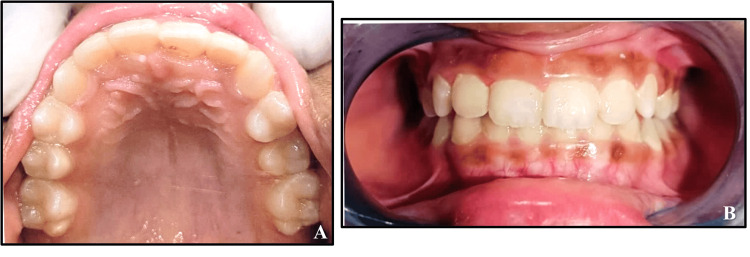
Post-treatment records (A) Post-treatment photograph of maxillary arch. (B) Post-treatment photograph at centric occlusion.

Case 2

A seven-year-old boy reported to the Department of Pediatric and Preventive Dentistry with a chief complaint of midline spacing in the upper jaw. The patient's medical and dental histories were non-contributory. The parent or the child reported no history of trauma. There were no significant findings in general and extraoral examinations. During the intraoral examination, it was observed that the patient was in a mixed dentition stage. There was a space of over 2 mm between the two central incisors. In soft tissue examination, the maxillary labial frenulum was found to be thick with attachment over the papilla between the two central incisors (Figure 3).

**Figure 3:**
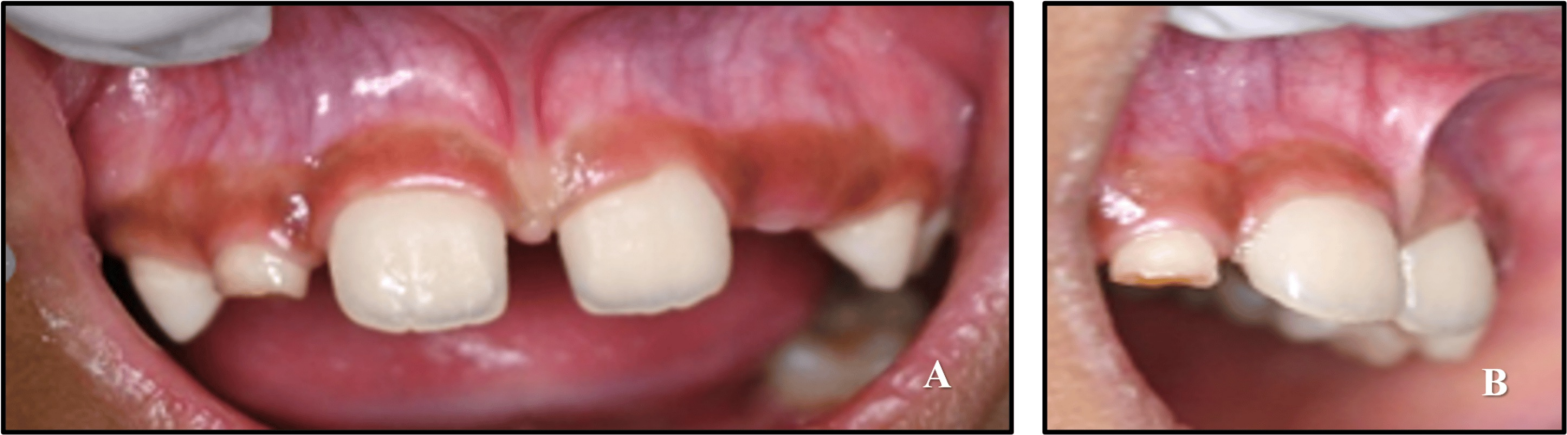
Pre-treatment intraoral photograph (A) Frontal view of maxillary labial frenum. (B) Lateral view of the maxillary labial frenum.

There was blanching on separating the upper lip. An intraoral periapical radiograph was suggested to search for the existence of any supernumerary teeth. Radiographic evaluation did not show any abnormality. Considering the functional aspects, the patient was a nasal breather with a normal swallowing pattern and tongue posture but an incompetent lip seal.

Treatment Plan

Treatment for high maxillary labial frenum: At the initial visit, informed consent was obtained, and complete oral prophylaxis and impression-taking of the upper and lower arches were performed. The patient was referred to the Department of Periodontology and Oral Implantology for assessment and treatment of the maxillary labial frenum. Written surgical consent from his parents was elicited before surgery. Under all aseptic precautions and conditions, the local anesthetic agent (2% lignocaine with adrenaline 1:80,000) was injected after applying topical lignocaine gel. For protection against laser radiation, the patient and the operator wore protective goggles before treatment.

Prior to the procedure, a 400 μm surgical laser tip was used. A 980 nm (EPIC X Diode Laser, Biolase, CA) tip was then used to vertically ablate the bottom of the labial frenum, and the frenectomy was performed (Figure 4). Relief of fibrous attachment from the labial frenum was followed by hemostasis without suturing (Figure 5).

**Figure 4:**
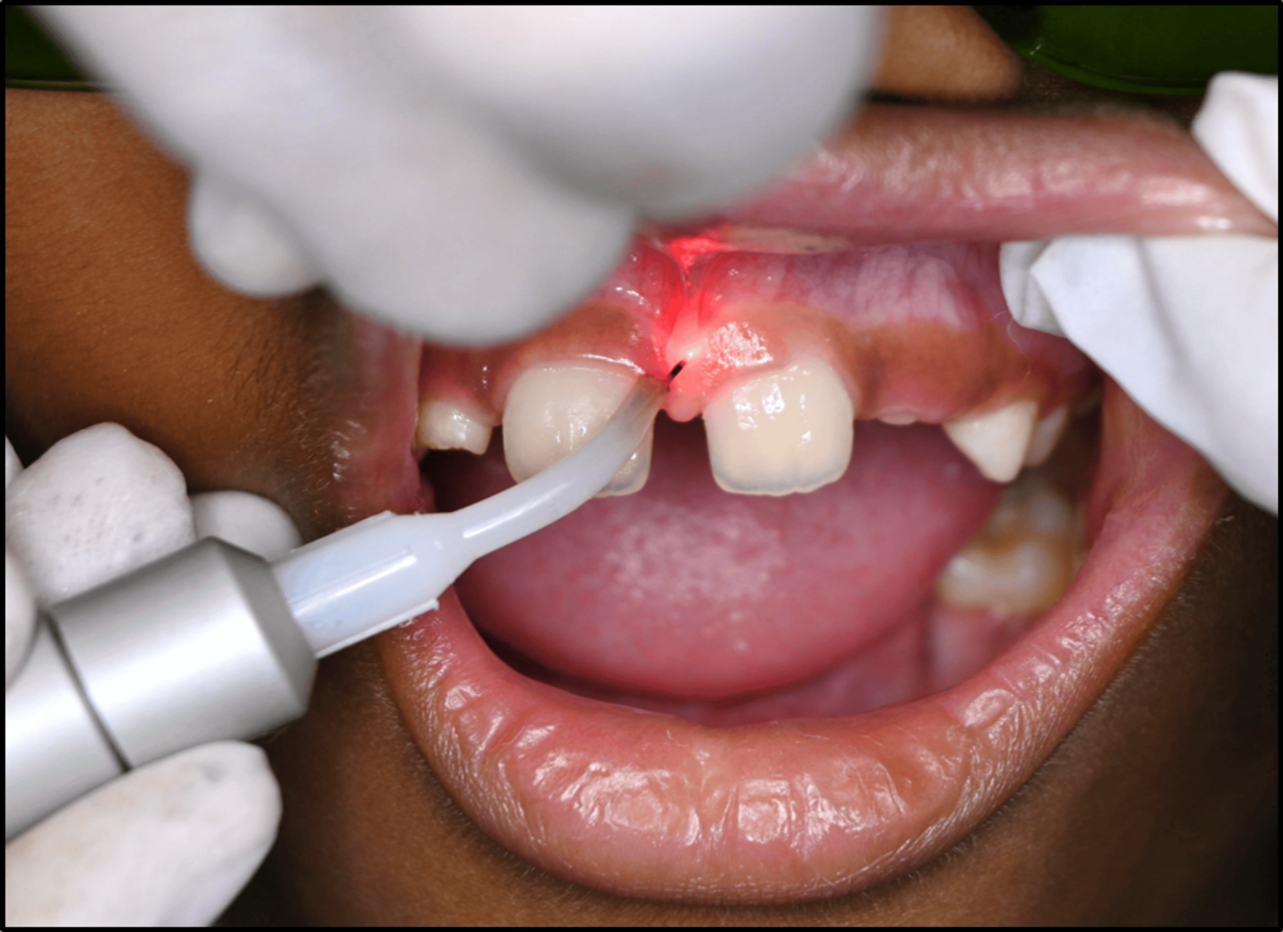
Intraoperative photograph during laser frenectomy

**Figure 5:**
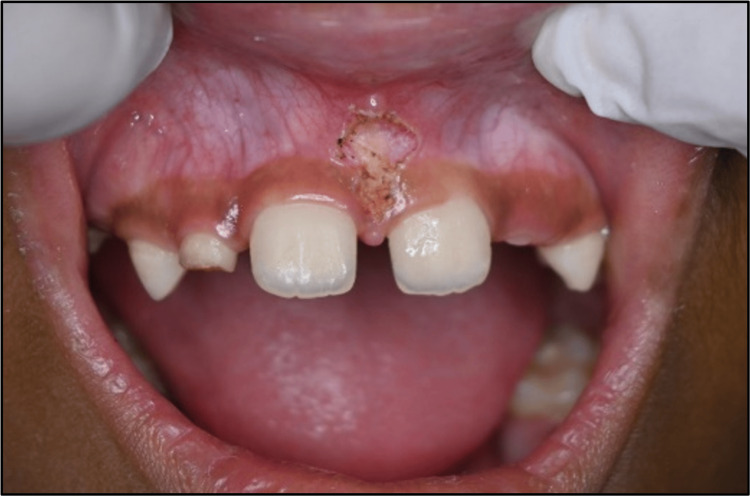
Immediate post laser frenectomy

In the postoperative period, the patient was provided with postoperative directions and analgesics. It was followed up after seven days and again after fourteen days, when sufficient healing was observed on examination (Figure 6). The patient was referred to the Pediatric and Preventive Dentistry department for further management.

**Figure 6:**
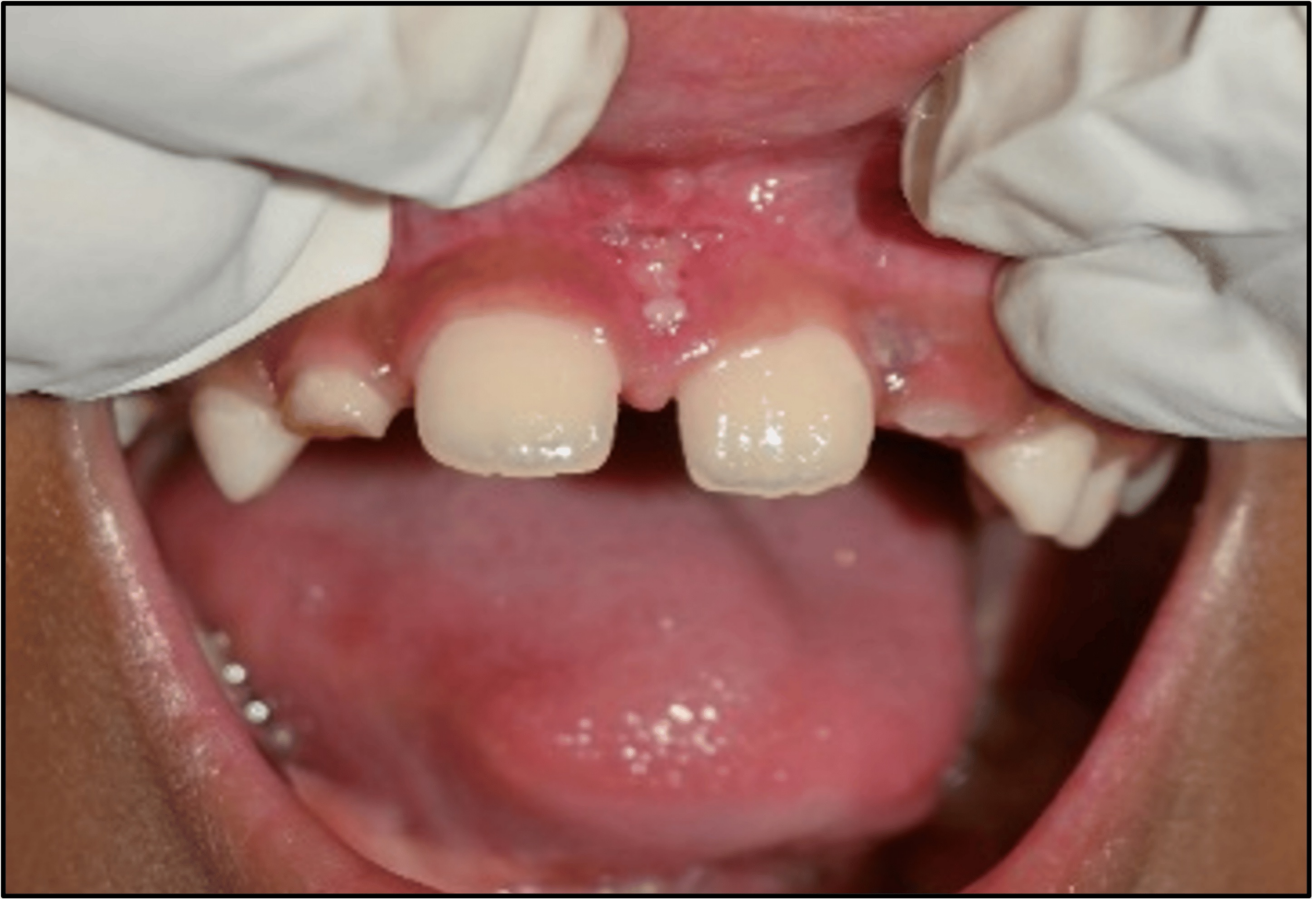
Seventh-day follow-up post laser frenectomy

Treatment for correction of single-tooth crossbite: Following uneventful healing of the frenectomy area, the alignment of the maxillary left central incisor was assessed (Figure 7). Tongue blade therapy was recommended to the patient with the aid of an ice cream stick. The patient was instructed on how to use the ice-cream stick and was advised to perform the same at home. The patient was recalled after a two-week follow-up (Figure 8). Following the two-week follow-up, the parent reported an interesting piece of information: the patient had initiated tongue blade therapy just four days ago, as they were apprehensive about seeing the dentist. Not starting late, there was some relief as the crossbite was now edge-to-edge with the lower left central incisor. For the patient's thought of self-motivation, he was instructed to go on with the tongue-blade therapy and have a mirror placed in front of him for another two weeks.

**Figure 7:**
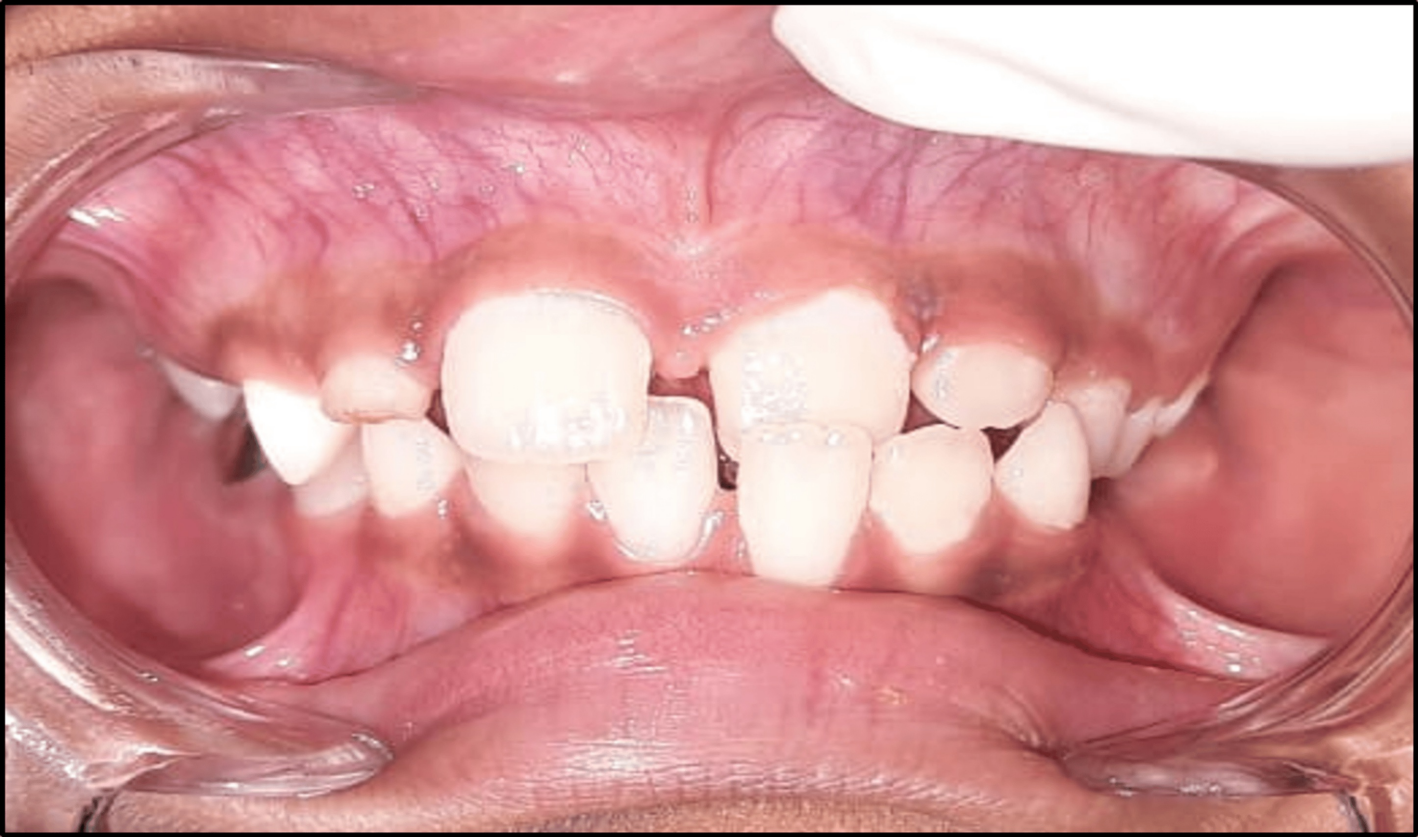
Position of 21 before tongue blade therapy

**Figure 8:**
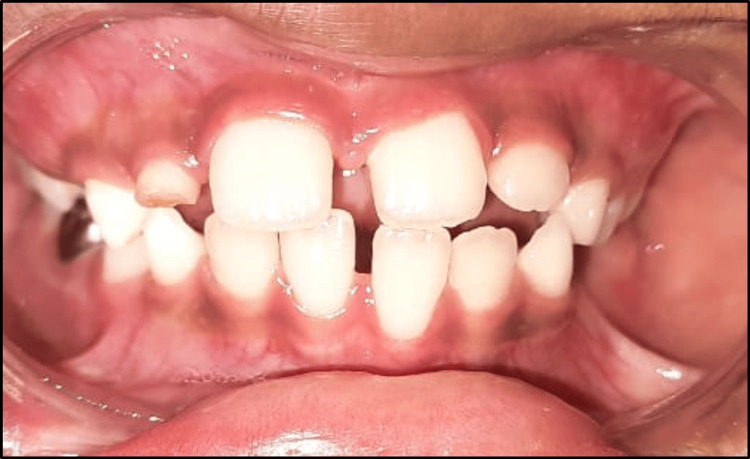
Two weeks after tongue blade therapy

The patient reported that after four weeks of tongue blade therapy, the crossbite had been corrected (Figure 9). The child was instructed to undergo a further six-month follow-up. During the six-month follow-up, the corrected tooth was found in position without any relapse. At the one-year follow-up, the corrected tooth was found to be in position without any relapse (Figure 10). The parents and the child were both satisfied with the result of the treatment given so far.

**Figure 9:**
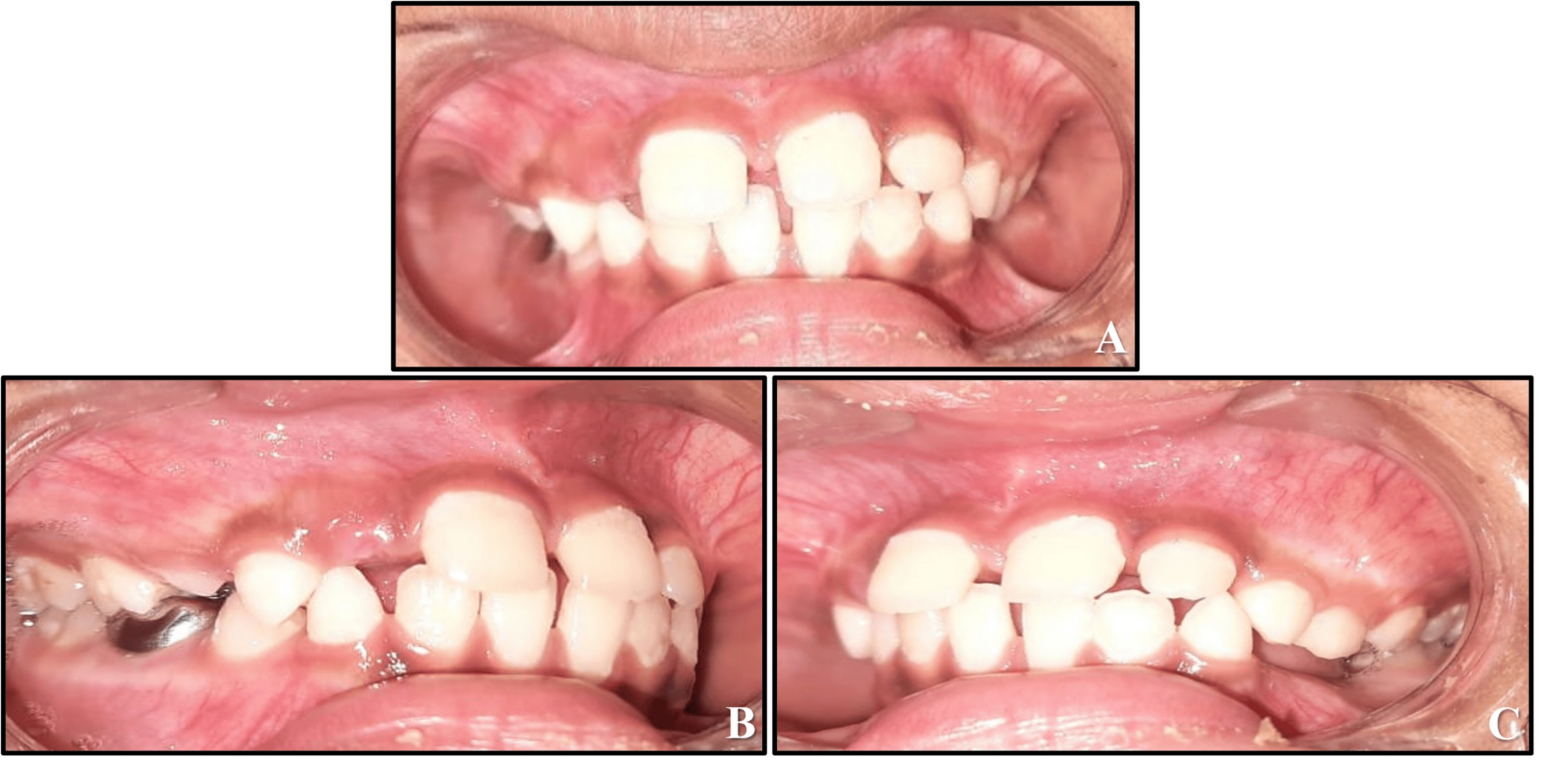
Four-week follow-up post tongue blade therapy (A) At centric occlusion. (B) At right lateral occlusion. (C) At left lateral occlusion.

**Figure 10:**
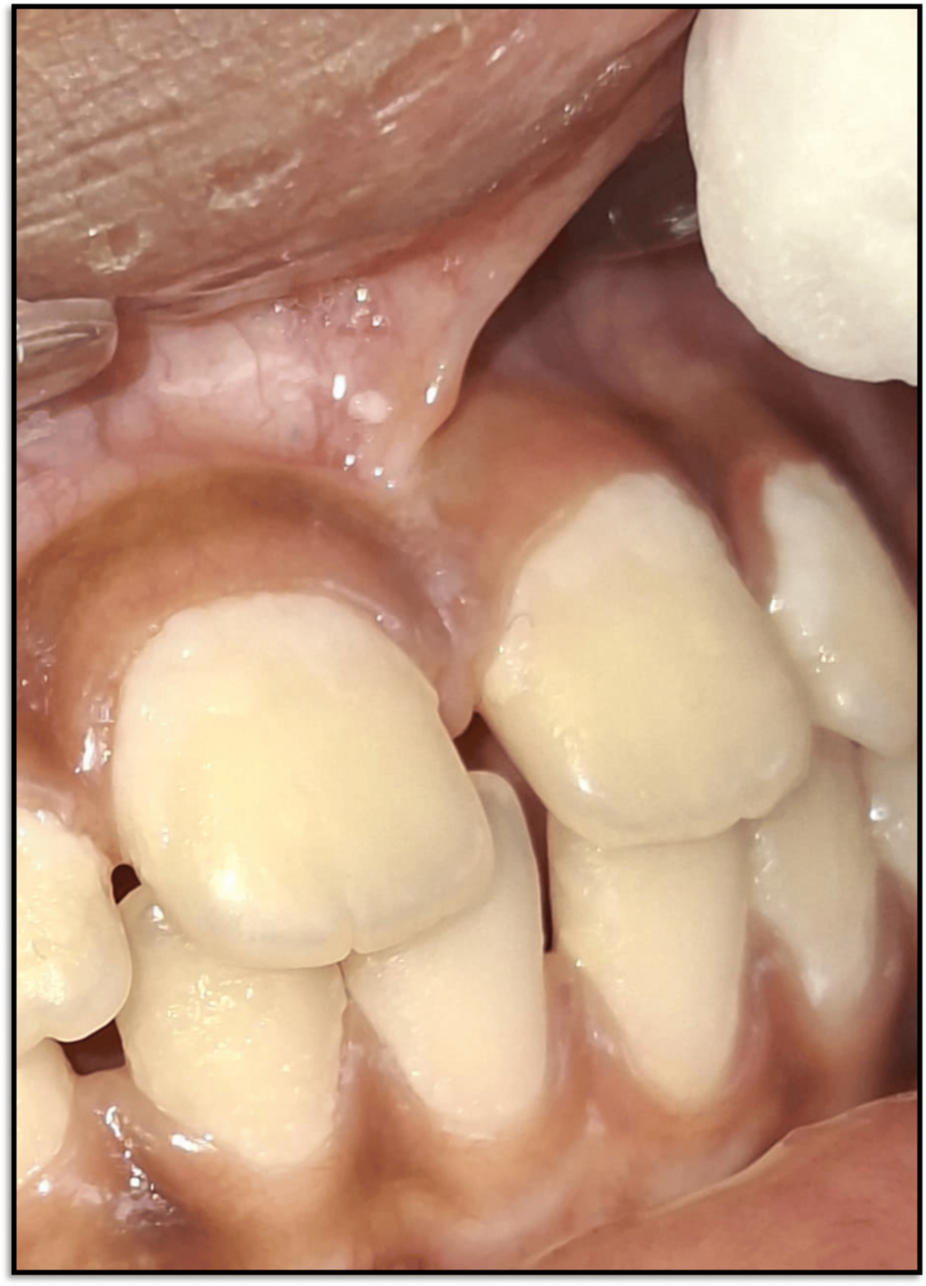
One year follow-up

Case 3

A nine-year-old male patient presented to the Department of Pediatric and Preventive Dentistry with the chief complaint of misaligned upper front teeth. His medical and family histories were non-contributory, and he had no evidence of any harmful oral habits. On intraoral examination, he was found to be in the mixed dentition phase with the maxillary left central incisor developing palatally, leading to an anterior crossbite. The palatal eruption was probably caused by the delayed exfoliation of the primary anterior teeth. The diagnosis of Angle's class I malocclusion with an anterior crossbite of tooth number 21 was made (Figure 11). Treatment alternatives were explained in detail to the patient's parents, and informed consent was received before initiating treatment.

**Figure 11:**
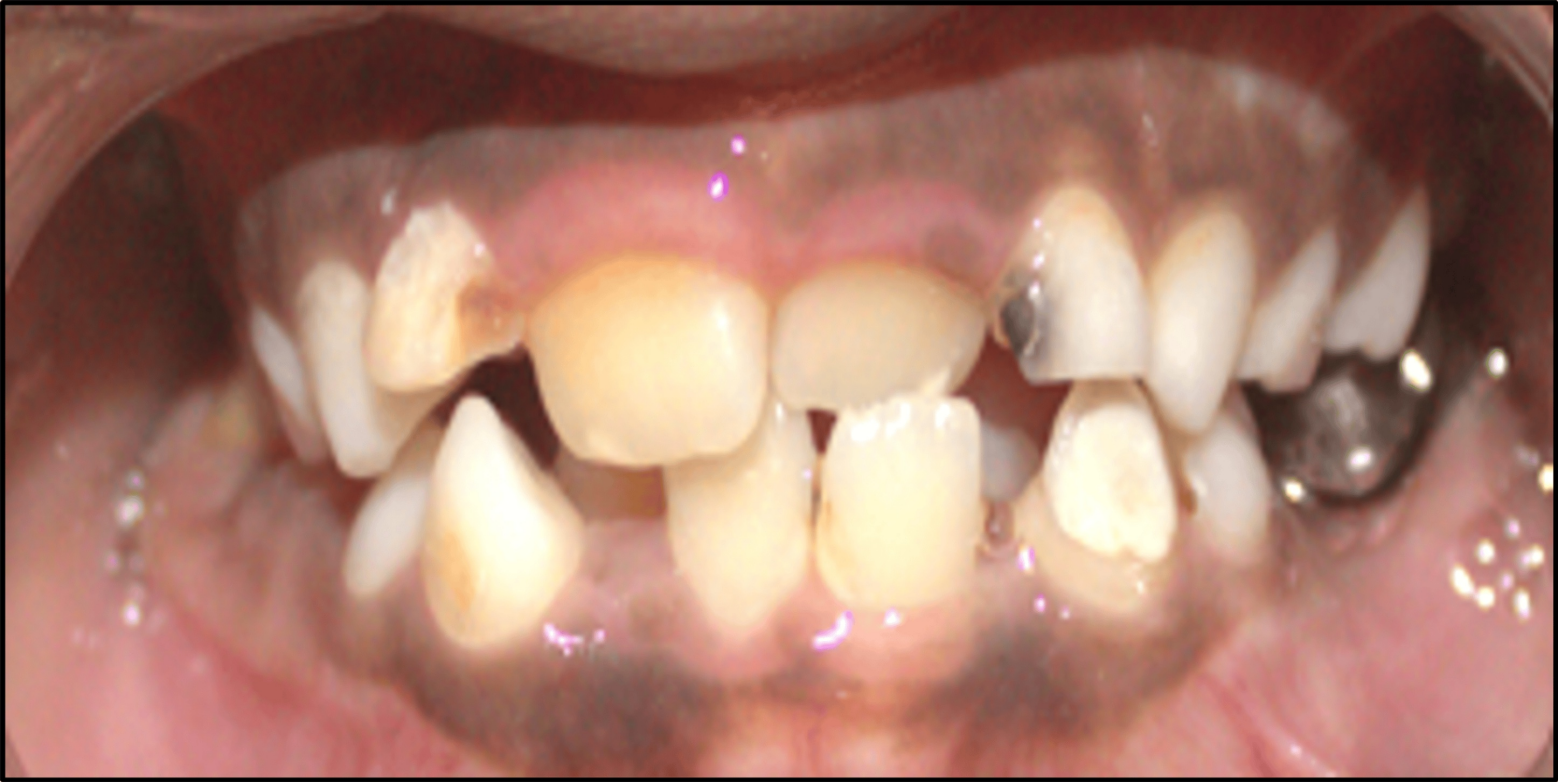
Pre-treatment photograph of developing crossbite with respect to 21

Treatment Plan

At the initial visit, following parental consent, pre-treatment records were collected in the form of intraoral and extraoral photographs, as well as upper and lower arch alginate impressions. At the follow-up visit, oral prophylaxis was performed. The patient was then instructed on tongue blade therapy as a non-surgical interceptive treatment to rectify the anterior crossbite. He was guided to position the ice cream stick between the left maxillary and left mandibular central incisors, bite hard to exert pressure for five seconds, release, and repeat 20 times, three times daily (Figure 12).

**Figure 12:**
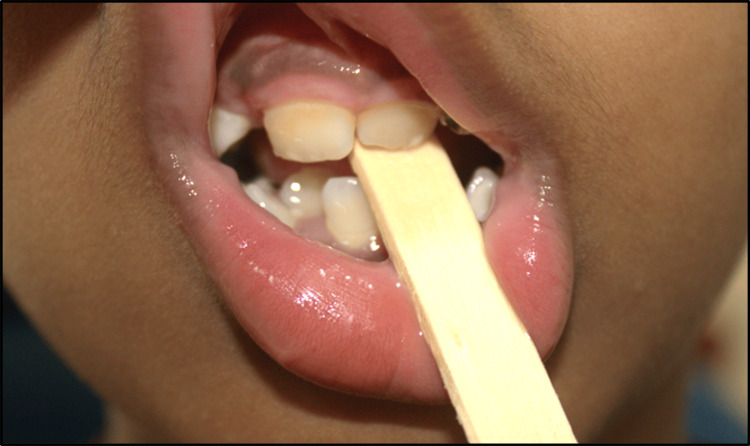
Placement of tongue blade

The parents were instructed to monitor the home therapy to guarantee compliance. No noticeable improvement was found at the one-week follow-up. The removal of the primary maxillary lateral incisors was done to allow for the eruption and proper alignment of the permanent incisors. The patient was also warned that if the crossbite persisted, he would have to wear a cumbersome appliance, such as a Hawley's appliance. With the fear of having to wear such appliance, the patient began to display enhanced compliance with the therapy using the tongue blade. At the subsequent follow-up, there was obvious correction of the crossbite (Figure 13).

**Figure 13:**
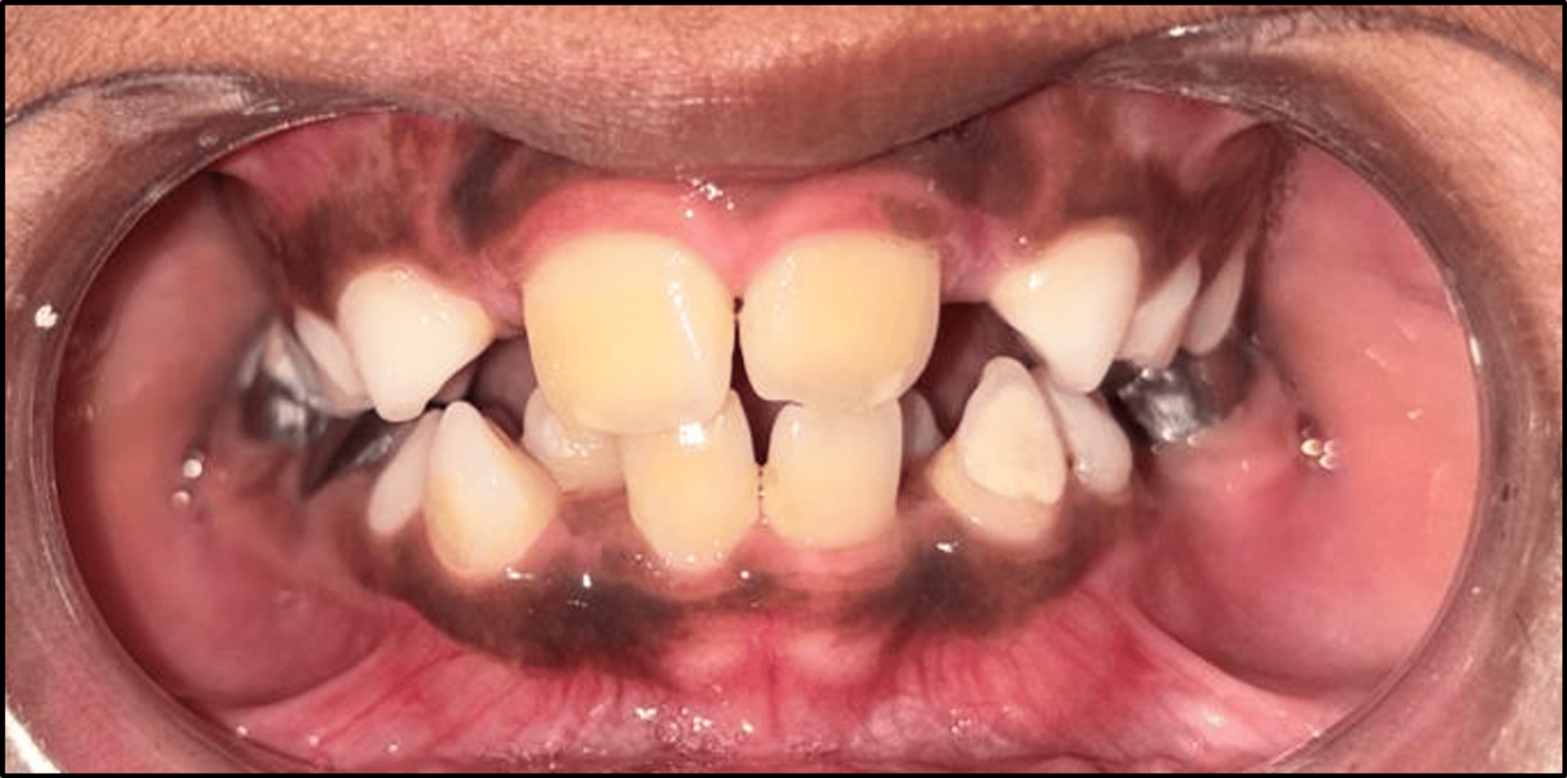
Four-week follow-up

## Discussion

Single-tooth anterior crossbite can be caused by several factors, including abnormal tooth eruption paths, retained primary teeth, trauma, or local space discrepancies. Most typically seen in the early mixed dentition stage, it can also be observed in various age groups. Treatment ranges from straightforward interceptive actions such as tongue blade therapy, Catlan's appliance, and spring removable plates to fixed sectional appliances, based on the cause and level of dental development. Accurate diagnosis and properly modulated interventions are crucial to achieving success.

Early orthodontic intervention to treat skeletal and dental discrepancies is referred to as "interceptive orthodontics." Early intervention prevents the need for complicated orthodontic treatment later on and encourages the patient to socialize, which may have been previously prohibited due to the patient's appearance and speech [11]. Ackerman and Proffit introduced the term "interceptive orthodontics" in 1980 to address issues related to developing dentition. Archaeological evidence in Egypt and ancient Rome revealed gold wires and bands as dental appliances. The concept of interceptive treatment was established during the mid-20th century, with the belief that early intervention may prevent the development of severe malocclusions in later years [12]. During the late 20th century, publications by Graber et al. further clarified the concept of interceptive orthodontics and its relevance. Principles of minimal intervention in interceptive orthodontics include early diagnosis, non-extraction strategies, modification of growth, natural eruption guidance, and patient cooperation [13]. Straub proposed the use of tongue blade therapy in his work. In this, he elaborated on the function and posture of the tongue in malocclusions, namely, anterior open bite and tongue thrusting, as well as stressing the importance of having nonsurgical and straightforward ways to retrain the lips and tongue. He also explained that the "suckling swallow" in infants progresses to a mature pattern with the child's growth and that retention of the infantile pattern results in resultant malocclusions. Abnormal swallowing is likely to cause dental malocclusions (such as flaring of incisors), future orthodontic problems, speech disorders, and muscle dysfunction [14]. An anterior crossbite is an abnormality that, on first identification, should be addressed.

It is not necessarily a self-correcting anomaly and needs specific intervention. Due to the etiological factors that contribute to crossbite occurrence, several factors are involved in its management, including correcting space adequacy, extracting supernumerary teeth, and performing a frenectomy (if necessary). Some of the least invasive options, such as bite resin (with restoration or a crown on molars), tongue blade therapy, and removable or fixed orthodontic appliances, are employed. In addition, the most significant consideration for any of the modalities mentioned above is the management of a child's behavior. Parents' and children's cooperation in adhering to these modalities is underrated. Other considerations, such as whether there is an "ugly duckling" stage, should also be taken into account. Crossbite correction depends on the choice of intervention and patient compliance. Based on the nature of the forces applied, various types of appliances can be used.

For heavy-intermittent forces, an acrylic inclined bite plane or tongue blade is recommended. For light-continuous forces, a removable appliance with auxiliary springs, a removable plate with a screw, or a fixed light arch wire is suitable. For those requiring skeletal correction, maxillary protraction devices or chin-cup therapy should be considered. Patient compliance is another crucial factor to consider. As young children, they have not yet developed to the level of cognition that allows them to understand why such appliances need to be used. In two instances, minimal interventional therapy with a tongue blade was applied, as it is a straightforward procedure that requires patient compliance, which was achieved through self-motivation using the mirror technique [15].

In one of the cases, because of the thick labial frenum having papillary-type frenulum attachment, maxillary labial frenectomy was done with a laser. It was preferred over electrocautery, as laser is a minimally invasive procedure, requiring less surgical time, with better patient cooperation and less postoperative discomfort while speaking and chewing [16]. A 980 nm diode laser was utilized, which is affordable, provides satisfactory control of bleeding, and promotes moderate healing. Strict guidelines should be established for laser use to determine which type of laser is suitable for a particular situation.

## Conclusions

This case report highlights the pivotal role played by individualized, minimally invasive strategies in treating anterior single-tooth crossbites in children during the mixed dentition stage. The initial case demonstrated that correction using a removable Hawley's appliance incorporating a Z-spring and posterior bite plate is feasible for correcting crossbites that require controlled active tooth movement, providing a predictable and patient-compliant solution. The second case underscored the need to detect and treat contributory etiological factors, such as high frenular attachment, before treatment, lest they undermine the chances of successful treatment. In this instance, a two-stage procedure involving frenectomy supplemented by tongue blade therapy was advantageous, reiterating the necessity for an in-depth assessment rather than appliance therapy alone. The third case demonstrated that, in appropriately selected patients, the early and regular application of simple techniques, such as tongue blade therapy alone, is capable of causing spontaneous correction without the need for active orthodontic appliances.

Together, these cases reaffirm that the key to effective management of anterior crossbite in the mixed dentition is timely diagnosis, appropriate etiological evaluation, and the choice of the most suitable, low-cost, and minimally invasive intervention best suited to the individual child. Such focused early treatment not only corrects the immediate issue but can also potentially prevent the worsening of malocclusion severity, saving future orthodontic treatment from being longer and more complex.

## References

[1] Vithanaarachchi SN, Nawarathna LS (2017). Prevalence of anterior cross bite in preadolescent orthodontic patients attending an orthodontic clinic. Ceylon Med J.

[2] Alaoui ML, Chhoul H (2019). Management of anterior single tooth crossbite in mixed dentition: a case report. IOSR J Dent Med Sci.

[3] Lo Giudice A, Polizzi A (2025). Pediatric treatment of anterior-upper-single dental crossbite using a versatile sagittal screw system: a case series. Pediatr Rep.

[4] Bayrak S, Tunc ES (2008). Treatment of anterior dental crossbite using bonded resin-composite slopes: case reports. Eur J Dent.

[5] Gahlod N (2023). Management of anterior single tooth crossbite using removable posterior teeth bite plane along with Z-spring: a case report. J Pharm Res Int.

[6] Koundal L, Priyanka K (2023). Management of anterior crossbite using Catlan’s appliance in the early mixed dentition period: a case report. Int J Adv Res.

[7] Gupta S, Vyas P, Sukhija U, Chauhan D, Nagi R (2023). Correction of single tooth anterior crossbite using sectional fixed orthodontic appliance: a case series. Univ J Dent Sci.

[8] Naji MR, Ababneh SH (2024). Orthodontic treatment and its impact on the overall wellness of pediatric patients in school settings: a review. J Cardiovasc Dis Res.

[9] Vishnu G, R R (2024). Comparative clinical outcomes in the treatment of anterior crossbite using three different appliances: a case series. Cureus.

[10] Gurusamy K, Os R, Krishna P T, R N (2011). Oral rehabilitation of an S-ECC case with orthodontic intervention: 18 months follow-up. Int J Clin Pediatr Dent.

[11] King GJ, Brudvik P (2010). Effectiveness of interceptive orthodontic treatment in reducing malocclusions. Am J Orthod Dentofacial Orthop.

[12] Xhemnica R, Rroço M (2022). Preventive and interceptive orthodontic treatment. Eur J Nat Sci Med.

[13] Graber TM, Vanarsdall RL, Vig KW (2005). Orthodontics: Current Principles and Techniques.

[14] Straub WJ (1960). Malfunction of the tongue: Part I. The abnormal swallowing habit: Its cause, effects, and results in relation to orthodontic treatment and speech therapy. Am J Orthod.

[15] Anindyanari A, Sinaredi BR, Nuraini P (2025). Timely intervention, lasting results: Managing dental anterior crossbite with a removable appliance. World J Adv Res Rev.

[16] Xie L, Wang P, Ding Y, Zhang L (2022). Comparative frenectomy with conventional scalpel and dual-waved laser in labial frenulum. World J Pediatr Surg.

